# PReS13-SPK-1576: Unmeet needs in JIA treatment

**DOI:** 10.1186/1546-0096-11-S2-I25

**Published:** 2013-12-05

**Authors:** N Ruperto

**Affiliations:** 1 Pediatria Ii, Reumatologia, Printo, Istituto G. Gaslini, Genova, Italy

## 

Conducting clinical trials in paediatric rheumatology has been difficult in the past mainly because of the lack of funding for academic studies and the lack of interest by pharmaceutical companies for the small and non-rewarding paediatric market. The situation changed dramatically few years ago with the introduction of the Best Pharmaceuticals for Children Act in USA and of a specific legislation for paediatric medicines development (Paediatric Regulation) in the European Union (EU).

The main reasons for success are: the availability of two large international non-for-profit networks working in close collaboration, such as the Pediatric Rheumatology Collaborative Study Group (PRCSG at http://www.prcsg.org), covering North America, and the Paediatric Rheumatology International Trials Organisation (PRINTO at http://www.printo.it), covering more than 50 countries worldwide; the availability of validated measures to evaluate response to therapy, now called JIA American College of Rheumatology (ACR) criteria, accepted by both the Food and Drug Administration (FDA) and the European Medicines Agency (EMA); last but not least the advent of the biologic therapies (anti TNF, Anti IL 1 and 6, anti CTL4Ig) which have revolutionized juvenile idiopathic arthritis (JIA) treatment.

Some problems however remain still to be solved: There is a need to harmonise all the regulatory aspects related to drugs that are used in the treatment of paediatric rheumatic diseases and in particular in JIA; the issue of me too drugs; the issue of proper pK studies; the ethics of drugs provision and of trial implementation; the implementation of proper pharmacovigilance systems.

This presentation will review the reasons for success and the problems that still remains to be solved for conducting trials in JIA.

## Disclosure of interest

None declared.

